# The acute glucose lowering effect of specific GPR120 activation in mice is mainly driven by glucagon-like peptide 1

**DOI:** 10.1371/journal.pone.0189060

**Published:** 2017-12-05

**Authors:** Linda Sundström, Susanna Myhre, Monika Sundqvist, Andrea Ahnmark, William McCoull, Piotr Raubo, Sam D. Groombridge, Magnus Polla, Ann-Christin Nyström, Lisbeth Kristensson, Mats Någård, Maria Sörhede Winzell

**Affiliations:** 1 Discovery Sciences, IMED Biotech Unit, AstraZeneca, Gothenburg, Sweden; 2 Cardiovascular and Metabolic Diseases, IMED Biotech Unit, AstraZeneca, Gothenburg, Sweden; 3 Medicinal Chemistry, IMED Biotech Unit, AstraZeneca, Alderley Park, United Kingdom; University of Ulster, UNITED KINGDOM

## Abstract

The mechanism behind the glucose lowering effect occurring after specific activation of GPR120 is not completely understood. In this study, a potent and selective GPR120 agonist was developed and its pharmacological properties were compared with the previously described GPR120 agonist Metabolex-36. Effects of both compounds on signaling pathways and GLP-1 secretion were investigated *in vitro*. The acute glucose lowering effect was studied in lean wild-type and GPR120 *null* mice following oral or intravenous glucose tolerance tests. *In vitro*, in GPR120 overexpressing cells, both agonists signaled through Gα_q_, Gα_s_ and the β-arrestin pathway. However, in mouse islets the signaling pathway was different since the agonists reduced cAMP production. The GPR120 agonists stimulated GLP-1 secretion both *in vitro* in STC-1 cells and *in vivo* following oral administration. *In vivo* GPR120 activation induced significant glucose lowering and increased insulin secretion after intravenous glucose administration in lean mice, while the agonists had no effect in GPR120 *null* mice. Exendin 9–39, a GLP-1 receptor antagonist, abolished the GPR120 induced effects on glucose and insulin following an intravenous glucose challenge. In conclusion, GLP-1 secretion is an important mechanism behind the acute glucose lowering effect following specific GPR120 activation.

## Introduction

With a prevalence of 415 million affected patients in 2015, type 2 diabetes is one of the most rapidly growing diseases worldwide (http://www.diabetesatlas.org) [1]. The major reasons for this is likely unhealthy diet and lifestyle choices leading to obesity, insulin resistance and elevated levels of free fatty acids in plasma [2]. Free fatty acids are categorized as short, medium, long and very long-chain, all of which can act as signaling molecules for a group of G-protein coupled receptors (GPCRs) consisting of GPR40, GPR41, GPR43, GPR84 and GPR120 [3–7]. GPR120, also known as the free fatty acid receptor 4 (FFAR4), has been suggested to act as a lipid sensor in the body, being involved in a diversity of processes like adipogenesis, inflammation and regulation of metabolic control [4, 8, 9].

Dysfunction in GPR120 signalling has been associated with development of obesity, where a mutation in the human GPR120 gene, inhibiting GPR120 signaling, has been observed in obese subjects [8]. GPR120 is activated by medium and long chain fatty acids, which leads to both β-arrestin recruitment and G-protein dependent signaling, by coupling to Gα_q_ [4, 10]. The different pathways have been suggested to be responsible for different biological functions [9, 11]. Studies have shown that GPR120 is expressed in multiple tissues including adipocytes, taste buds and certain immune cells possibly mediating insulin-sensitizing and anti-inflammatory effects [9]. GPR120 is also expressed in enteroendocrine cells in the gut, where it is suggested to mediate fatty acid stimulated release of glucagon-like peptide-1 (GLP-1) [4, 12]. GLP-1 is an incretin hormone with many biological functions including potentiation of glucose-stimulated insulin secretion (GSIS), resulting in decreased plasma glucose levels [13, 14]. GLP-1 receptor agonists and DPP4 inhibitors, which reduce the degradation of endogenous GLP-1, are commonly used for treatment of type 2 diabetes [15], and this concept may be further expanded together with GLP-1 secretagogues. GPR120 mediated release of GLP-1 was previously shown in clonal enteroendocrine cells with α-linoleic acid (ALA) [4] and a small molecule GPR120 agonist TUG-891 [16], but has been challenged by Paulsen et al [17] who stated that GPR120 does not play a major role in the regulation of GLP-1 secretion. These contradicting results led us to investigate the mechanism of action of GPR120 activation with respect to GLP-1 release. Medium and long chain fatty acids activates both GPR120 and GPR40 [4, 6, 18] and previous studies exploring the biological role of GPR120 have used fatty acids such as ALA or small molecule non-selective GPR120 agonists. In this study we have developed a novel GPR120 selective agonist (AZ13581837) and compared it with a previously described agonist, Metabolex-36 [19] on GLP-1 secretion in lean mice.

## Material and methods

### GPR120 agonists

**Fig 1:**
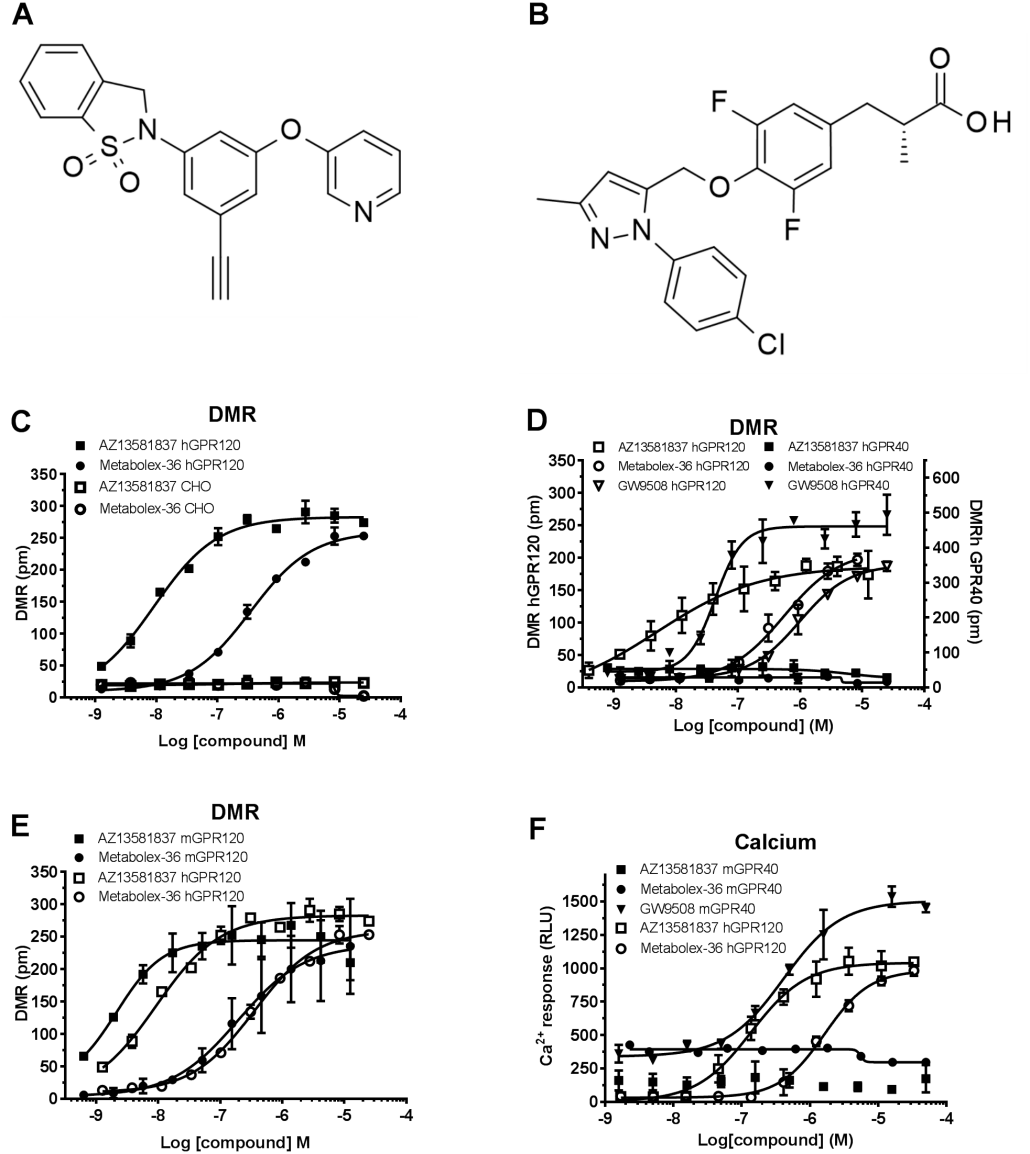
Structure of AZ13581837 and Metabolex-36 and specificity of the compounds for human and mouse GPR120 and human or mouse GPR40. **A)** Chemical structure of AZ13581837 and **B)** Metabolex-36.**C)** Effect of AZ13581837 (squares) and Metabolex-36 (circles) on DMR response in CHO-hGPR120 (filled symbols) and CHO (open symbols). **D)** Activity in CHO-GPR40 cells for AZ13581837 (filled squares), Metabolex-36 (filled circles) and GW9508 (filled triangles). Activity in CHO-hGPR120 cells is shown as reference for AZ13581837 (open squares), Metabolex-36 (open circles) and GW9508 (open triangles). **E)** Cross species selectivity evaluated in CHO-mGPR120 cells using a DMR assay. Activity of AZ13581837 (squares) and Metabolex-36 (circles) on mouse GPR120 (filled symbols) compared to human GPR120 (open symbols). **F)** Cross species selectivity for GPR40 evaluated using a calcium mobilization assay. Effect of AZ13581837 (filled squares) and Metabolex-36 (filled circles) on mouse GPR40 with GW9508 (filled triangles) as reference. Activity in CHO-hGPR120 cells is shown as comparison for AZ13581837 (open squares) and Metabolex-36 (open circles). Data are shown as mean ± SEM run in duplicates or more and representative for two or more independent experiments.

The two GPR120 agonists used in this study were the newly developed AZ13581837 (2-(3-ethynyl-5-(3-pyridyloxy)phenyl)-3H-1,2-benzothiazole 1,1-dioxide) and Metabolex-36 (3-(3,5-difluoro-4-((3-methyl-1-phenyl-1H-pyrazol-5-yl)methoxy)phenyl)-2-methylpropanoic acid) [20] (Fig 1A and 1B). AZ13581837 was synthesized according to the protocol (S1 Appendix). For *in vitro* experiments stock dilutions of the compounds were prepared in DMSO. For calcium mobilization and dynamic mass redistribution (DMR) assays compounds were pre-diluted in Hanks´ buffered saline solution (HBSS), 20 mM HEPES and 0.01% bovine serum albumin (BSA) (1% DMSO). For *in vivo* experiments, compounds were formulated in 0.5% hydroxypropyl methycellulose (HPMC) and 0.1% Tween-80.

### Cell lines

The short isoform of human GPR120 (361 amino acids) was stably expressed in Chinese hamster ovary (CHO K1) cells (ATCC) and the mouse GPR120 was subcloned in pCDN5/FRT vector and stably expressed in CHO K1 cells (ATCC), referred to as CHO-hGPR120 and CHO-mGPR120 respectively. Control cells expressing the empty vector are referred to as CHO. The Tango^™^ GPCR Assay System cells were custom generated U2OS expressing human GPR120 (short isoform, Swissprot Q5Nul3-2) by Life Technologies, referred to as U2OS-hGPR120. Human GPR40 (Genebank accession number BC120944.1) was stably expressed in CHO K1 (ChanTest Corp, Cleveland, OH) and mouse GPR40 (silent SNP at position 779, A instead of G) was subcloned into pIRESneo3 and stably expressed in HEK293 cells (ATCC), referred to as CHO-hGPR40 and HEK293-mGPR40 respectively. STC-1 cells (ATCC), a mouse enteroendocrine cell line, were used for GLP-1 secretion.

### Calcium mobilization assay

CHO-hGPR120 and CHO cells were cultured in DMEM/F-12 (Invitrogen) with 10% FBS (Sigma) and HEK293-mGPR40 in DMEM (Invitrogen) supplemented with 10% FBS in 384-well poly-D-Lysin coated microplates (Greiner) at 37°C, 95% humidity and 5% CO_2_. After 19 hours cell media was replaced with Calcium4 dye (Molecular Devices) in HBSS (Invitrogen), 20 mM HEPES (Invitrogen), pH 7.4 and 1.25 mM Probenecid (Sigma). After 1 hour at room temperature agonists were added and calcium mobilization was detected as change in fluorescence (λ_ex_ = 488 and λ_em_ = 540 nm) using a FLIPR^TETRA^ (Molecular Devices).

### β-arrestin assay

U2OS-hGPR120 cells cultured in Freestyle medium (Invitrogen) were incubated for 5 hours with GPR120 agonists in 384-well Cell BIND plates (Corning) at 37°C, 95% humidity and 5% CO_2_. LiveBLAzer^™^ FRET B/G working solution (Invitrogen) was added and after 2 hours at room temperature fluorescence intensity (λ_ex_ = 405 nm, λ_em_ = 530 and 460 nm) was detected using a Pherastar FS (BMG Labtech) according to the manufacturers protocol.

### DMR assay

DMR (dynamic mass redistribution) was measured by resonant waveguide grating (RWG) using an Epic biosensor (Corning) [21]. CHO-hGPR120, CHO-mGPR120 and CHO cells in DMEM/F-12 (Invitrogen) were cultured in fibronectin coated 384-well Epic plates (Corning) at 37°C, 95% humidity and 5% CO_2_ for 5 hours with 10% FBS (Sigma), followed by 19 hours without FBS. CHO-hGPR40 cells were cultured at 37°C, 95% humidity and 5% CO_2_ for 20–24 hours in Ham’s F-12 Nutrient Mix with GlutaMAX (Invitrogen) supplemented with Non-Essential Amino Acids (Gibco) and 10% FBS (Sigma) in fibronectin coated 384-well Epic plates (Corning). Following the incubations culture media was replaced with HBSS (Invitrogen), 20 mM HEPES (Invitrogen), pH 7.4 and 1% DMSO and equilibrated to 26°C in the Epic for thirty min before addition of agonists and detection of DMR.

To explore the G-protein signaling pathways, cells were treated 16–20 hours with 100 ng/mL pertussis toxin (PTX, Sigma), to inhibit activation of Gα_i_, or 1 mg/mL of cholera toxin (CTX, Sigma), to inhibit Gα_s_ activation. For inhibition of Gα_q_, cells were treated for 1 hour with 1 μM of Gα_q_ inhibiting component (QIC, Institute of Pharmaceutical Biology, University of Bonn) [22].

### cAMP assay

CHO-hGPR120 or CHO cells in HBSS (Invitrogen), 20 mM HEPES (Invitrogen), pH 7.4, 0.01% BSA, 1.5 mM 3-isobutyl-1-methylxanthine (Sigma) and d2-labeled cAMP (CisBio) were incubated with GPR120 agonists (1% DMSO) for 45 min at room temperature in white small-volume 384-well plates (Greiner). The reaction was terminated by addition of conjugate and lysis buffer (CisBio) supplemented with anti-cAMP Cryptate (CisBio).

Mouse islets were isolated from pancreas by collagenase digestion, handpicked under microscope following multiple washing steps and dispersed to single cells using TrypLE Express (Gibco). The dispersed islet cells (20 000 cells/well) were incubated with the GPR120 agonists for 30 min in 96-well black plates (Corning) at 37°C in Krebs ringer phosphate Hepes buffer, pH 7.4, with 11 mM Glucose, 0.1% BSA and 0.5 mM 3-isobutyl-1-methylxanthine followed by addition of d2-labeled cAMP and anti-cAMP Cryptate in conjugation and lysis buffer (Cisbio). Produced cAMP was detected with homogenous time resolved fluorescence (HTRF) (λ_ex_ = 340 nm, λ_em_ = 665 and 615 nm) using a Pherastar (BMG Labtech).

### GLP-1 secretion

STC-1 cells were cultured in DMEM (Invitrogen) supplemented with 10% horse serum (ThermoFisher Scientific), 2.5% FBS (Hyclone) and 1% Penicillin/Streptomycin in 96-well poly-D-Lysine coated plates to 80–90% confluence at 37°C, 5% CO_2_. Cells were washed and pre-incubated in assay buffer (HBSS with Ca^2+^ and Mg^2+^ containing 20 mM HEPES, 0.1% BSA (Sigma) and 20 nM sitagliptin (dipeptidyl peptidase 4 (DPP-4) inhibitor), for 30 min in 37°C. GPR120 agonists were added and cells were incubated for 2 hours in 37°C. DMSO (0.1%) was used as control. GLP-1 secretion was measured in the supernatants (GLP-1 active, Millipore) by fluorescence detection. Concentration of active GLP-1 was derived by interpolation from a GLP-1 standard curve.

### Animals

Eight-week old female C57BL/6JOlaHSd (Harlan, Germany) or C57Bl/6 (Charles River, Germany) and male C57BL/6J (Jackson Laboratory, USA) mice were used for *in vivo* studies. GPR120 *null* mice were generated as described by Bjursell et al [23]. The animals were maintained in a temperature-controlled room (22°C) on a 12-hour light-dark cycle (lights on at 06:00 am) and were allowed to acclimatize for one week after arrival. The mice were housed in plastic cages with wooden bedding and nesting material in groups of five individuals with free access to lab chow (R70, Lantmännen, Sweden) and tap water. The body weight for female lean, eight-week old female C57BL/6JOlaHSd mice were between 20–25 g. Body weight for the female GPR120 *null* mice averaged 22.5±0.7 g (n = 8) and wildtypes 23.0±0.8 (n = 8). Male GPR120 *null* mice weighed 26.3±0.8 g (n = 10) and wild types 27.5±0.7 g (n = 10) at the time of the experiment. Blood samples were taken at indicated time points during the glucose tolerance tests from the tail vein. In the IVGTT, glucose was injected in one tail vein and blood samples were taken in another tail vein. In all glucose tolerance tests, mice were randomized into the treatment groups on their body weight. Weight matched groups were dosed in a randomized order alternating between vehicle and compound treatment. The dosing was performed in intervals to allow all mice to be dosed with glucose at exactly the same time after dose with compounds or vehicle. Blood samples were analysed in the same randomized order as the animals were dosed. After the experiments, the mice were anesthetized with isoflurane and after bleeding the mice for preparation of termination plasma samples, the mice were sacrificed by neck dislocation.

Principles of laboratory animal care were implemented according to Swedish and British authorities, and studies were approved by the Animal Ethics Committee in Gothenburg, Sweden or the British Home Office Animal Scientific Procedures Act 1986, respectively.

### Oral glucose tolerance test (OGTT) in healthy mice

OGTT tests were performed in lean male mice (body weight 27-32g), randomized on body weight, fasted for 16 hours. An oral glucose load (2 g/kg) was administered 30 min (AZ13581837, 7 and 18 mg/kg) or 60 min (Metabolex-36, 10, 30 and 100 mg/kg) after oral dosing with the GPR120 agonists and blood glucose was measured at -30, 0, 10, 25, 40, 60 and 90 min post glucose administration. The specificity of Metabolex-36 was examined in male GPR120 *null* mice. The administration time for the GPR120 agonists was optimized depending on the pharmacokinetic profile of each compound, in order to maximize the plasma exposure of the compounds during the experiments.

### Intravenous glucose tolerance test (IVGTT) in healthy mice

Lean female mice (body weight 20–25 g), were randomized on body weight and fasted 4 hours prior to the IVGTT. Metabolex-36 (100 mg/kg) was administered orally 60 min and AZ13581837 (35mg/kg) 30 min before the IVGTT (0.35 mg glucose/kg). Control mice were given vehicle (0.5% HPMC, 0.1% Tween-80) at corresponding time points. The specificity of AZ13581837 was examined in IVGTT in GPR120 *null* mice. GPR120 agonist doses were selected to reach plasma levels of at least 10 times over the *in vitro* EC_50_ value to achieve maximal activation of GPR120 (S2 Appendix). Thirty minutes prior to the glucose load, the mice were anaesthetised (20 mg/kg fluanisone/0.8 mg/kg fentanyl (Hypnorm^®^, Janssen, Beerse, Belgium) and 10 mg/kg midazolam (Dormicum^®^, Hoffman-LaRoche, Basel, Switzerland)) to allow for rapid blood sampling. The mice were kept at a heating pad to maintain body temperature.

In separate experiments, mice were pretreated with the GLP-1 receptor antagonist exendin 9–39 (30 nmol/kg, Bachem; intraperitoneally) alone or in combination with the GPR120 agonists, 15 min prior to the IVGTT. Insulin and glucose were measured 0, 1, 5, 10, 20 and 40 min after intravenous (iv) glucose administration. In separate groups of mice total GLP-1 levels were measured 60 min after oral administration of Metabolex-36 or 30 min after AZ13581837 and in corresponding vehicle (0.5% HPMC, 0.1% Tween-80) dosed mice. Blood samples were collected in EDTA coated tubes, centrifuged at 10000xg for 4 min, and plasma was separated and stored frozen until analyzed.

### Biochemical analysis

Glucose was measured using a glucometer (AccuCheck, Bayer). Circulating hormones were measured using specific ELISA kits; insulin (ultra-sensitive mouse insulin, Crystal Chemistry), and total and active GLP-1 (Millipore).

### Calculations and statistical analysis

*In vitro* data for ascending compound concentrations were analyzed with a 4-parameter logistic fit using the equation *y = A + ((B-A)/1 + ((C/x)^D)))* where A is no activation, B is full activation, C is the EC_50_ and D is the Hill slope using GraphPad Prism 6.

For OGTT a turnover model with a log-linear drug function [24] was used to model the glucose lowering effect determined as the effective concentration resulting in a 50% decrease in blood glucose over time (Ce_50_) compared to vehicle. All analyses were performed using Phoenix WinNonlin 6.4 (Certara, Princeton, NJ, USA). The area under the glucose excursion curve was calculated and used for statistical evaluation.

In the IVGTT, the acute insulin response (AIR) was calculated as the mean of suprabasal 1 and 5 min values, and the glucose elimination was quantified using the glucose elimination constant (K_G_) calculated as the slope of the logarithmic transformation of circulating glucose between 1 and 20 min after the glucose bolus. Multiple comparisons between the different groups were performed by one-way ANOVA. Tukey’s or Bonferroni’s post hoc test were used to calculate statistical differences between the groups. Significant statistical difference was considered at *p*<0.05. Data are presented as mean ± SEM.

## Results

### AZ13581837 is a GPR120 selective agonist

The newly developed AZ13581837 (Fig 1A) was compared with Metabolex-36 (Fig 1B) and both compounds were found to stimulate a DMR response in CHO-hGPR120 cells without activity in control CHO cells (Fig 1C), AZ13581837 being about 100-fold more potent than Metabolex-36 (Table 1). Neither AZ13581837 nor Metabolex-36 were able to induce a DMR response in CHO-hGPR40 cells (Fig 1D). As expected, GW9508 showed activity on both human GPR40 and GPR120, with EC_50_ 48 ± 0.7 nM (n = 9) and 0.77 ± 0.09 μM (n = 24), respectively (Fig 1D). AZ13581837 and Metabolex-36 were able to activate mouse GPR120 in DMR with EC_50_ of 4.3 ± 0.6 nM (n = 10) and 130 ± 20 nM (n = 2) respectively (Fig 1E), but did not activate mouse GPR40 in a calcium mobilization assay (Fig 1F). For reference GW9508 was included and activated mouse GPR40 with EC_50_ 0.9 ± 0.3 μM (n = 10) (Fig 1F). Thus both AZ13581837 and Metabolex-36 were selective for human and mouse GPR120 without activity on GPR40.

**Table 1:** *In vitro* pharmacology of AZ13581837 and Metabolex-36.

Compound	Calcium mobilization EC_50_ (nM)	DMR EC_50_ (nM)	cAMP EC_50_ (nM)	β-arrestin EC_50_ (nM)
**AZ13581837**	120 ± 20 (n = 5)	5.2 ± 0.6 (n = 34)	60 ± 7.0 (n = 3)	5.2 ± 0.4 (n = 9)
**Metabolex-36**	1800 ± 190 (n = 3)	570 ± 150 (n = 6)	6700 ± 1000 (n = 3)	1400 ± 700 (n = 3)

Potencies for agonists in calcium mobilization, DMR response and cAMP production in CHO-hGPR120 cells and β-arrestin recruitment in U2OS-hGPR120 cells. Data are mean ± SEM based on three or more independent experiments as indicated in the table.

### GPR120 agonists induce Gα_q_ and Gα_s_ signaling in CHO-hGPR120 cells

**Fig 2:**
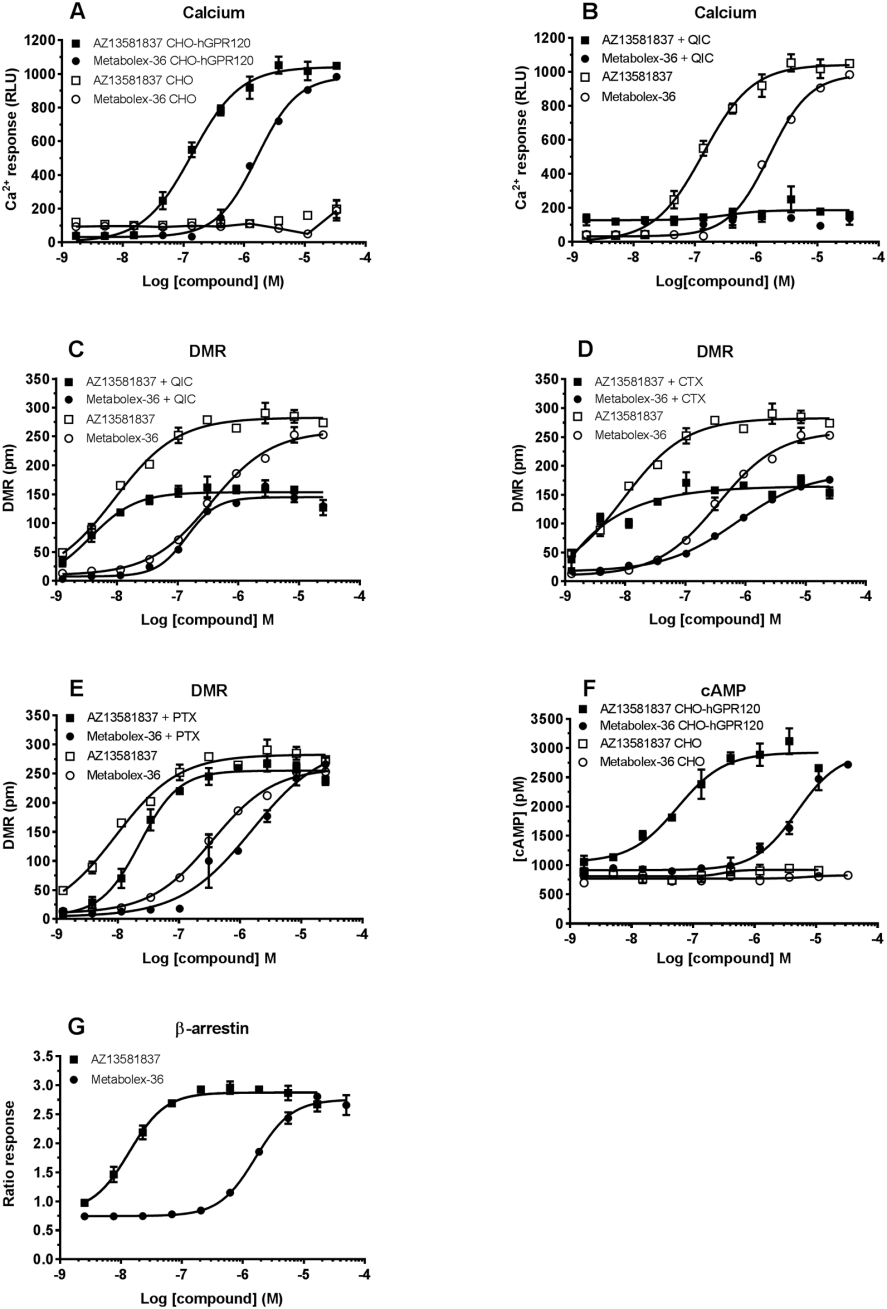
AZ13581837 and Metabolex-36 induce GPR120 dependent calcium mobilization, DMR response and cAMP production in GPR120 overexpressing cells. **A**) Effect of AZ13581837 (squares) and Metabolex-36 (circles) on calcium mobilization in CHO-hGPR120 (filled symbols) and CHO cells (open symbols). **B**) Calcium mobilization induced by AZ13581837 (squares) and Metabolex-36 (circles) in untreated (open symbols) and QIC treated (filled symbols) CHO-hGPR120 cells. **C**) DMR response from stimulation with AZ13581837 (squares) and Metabolex-36 (circles) in untreated (open symbols) and QIC treated (filled symbols) CHO-hGPR120 cells. **D**) DMR response from AZ13581837 (squares) and Metabolex-36 (circles) in untreated (open symbols) and CTX treated (filled symbols) CHO-hGPR120 cells. **E**) DMR response from stimulation with AZ13581837 (squares) and Metabolex-36 (circles) in untreated (open symbols) and PTX treated (filled symbols) CHO-hGPR120 cells. **F**) Effect of AZ13581837 (squares) and Metabolex-36 (circles) on cAMP production in CHO-hGPR120 (filled symbols) and CHO cells (open symbols). **G)** Recruitment of β-arrestin in U2OS-hGPR120 cells induced by AZ13581837 (filled squares) and Metabolex-36 (filled circles). Data are mean ± SEM of experiments run in duplicates or more and representative for two or more independent experiments.

Both GPR120 agonists selectively induced a calcium response in CHO-hGPR120 cells with no response in control CHO cells (Fig 2A). AZ13581837 was more potent compared to Metabolex-36 with EC_50_ 120 ± 20 nM and 1800 ± 190 nM, respectively (Table 1). The calcium mobilization induced by AZ13581837 and Metabolex-36 was completely abolished by pre-treating the CHO-hGPR120 cells with 1 μM of QIC [21], demonstrating the calcium response to be completely Gα_q_ dependent (Fig 2B). To explore whether activation of GPR120 involves other G-proteins, a holistic label free dynamic mass redistribution (DMR) assay, capable of detecting signaling by all G-proteins, was used. The DMR response was GPR120 specific since no activity was detected in parental CHO cells (Fig 1C). Unlike the calcium response, the GPR120 dependent DMR response was only partly reduced by the Gα_q_ inhibitor QIC (Fig 2C), indicating signaling by additional G-proteins. Indeed, inhibition of Gα_s_ by treating CHO-hGPR120 cells with CTX prior to addition of agonists, partly blocked the DMR response, suggesting GPR120 also to couple to Gα_s_ (Fig 2D). However, treating CHO-hGPR120 cells with PTX to inhibit Gα_i_ did not result in any significant decrease of the agonists’ ability to induce a DMR response (Fig 2E), suggesting no Gα_i_ signaling in these cells.

### GPR120 activation stimulated cAMP production in GPR120 over-expressing cells

Both AZ13581837 and Metabolex-36 caused a concentration dependent increase in cAMP levels in CHO-hGPR120 cells, and no increase was observed in control CHO cells (Fig 2F), confirming a GPR120 dependent cAMP production, with EC_50_ of 60 ± 7.0 nM for AZ13581837 and 6700 ± 1010 nM for Metabolex-36 (Table 1).

### GPR120 agonists recruit β-arrestin

GPR120 has been demonstrated to activate the non-G-protein dependent β-arrestin pathway involved in the internalization of the receptor [25]. We therefore evaluated β-arrestin in U2OS-hGPR120 cells using a Tango GeneBlazer assay. Both AZ13581837 and Metabolex-36 efficiently recruited β-arrestin upon activation of GPR120 with EC_50_ of 5.2 ± 0.4 nM and 1400 ± 700 nM respectively (Fig 2G, Table 1).

### GPR120 agonists inhibit cAMP production in mouse islets and increase GLP-1 secretion from STC-1 cells

**Fig 3:**
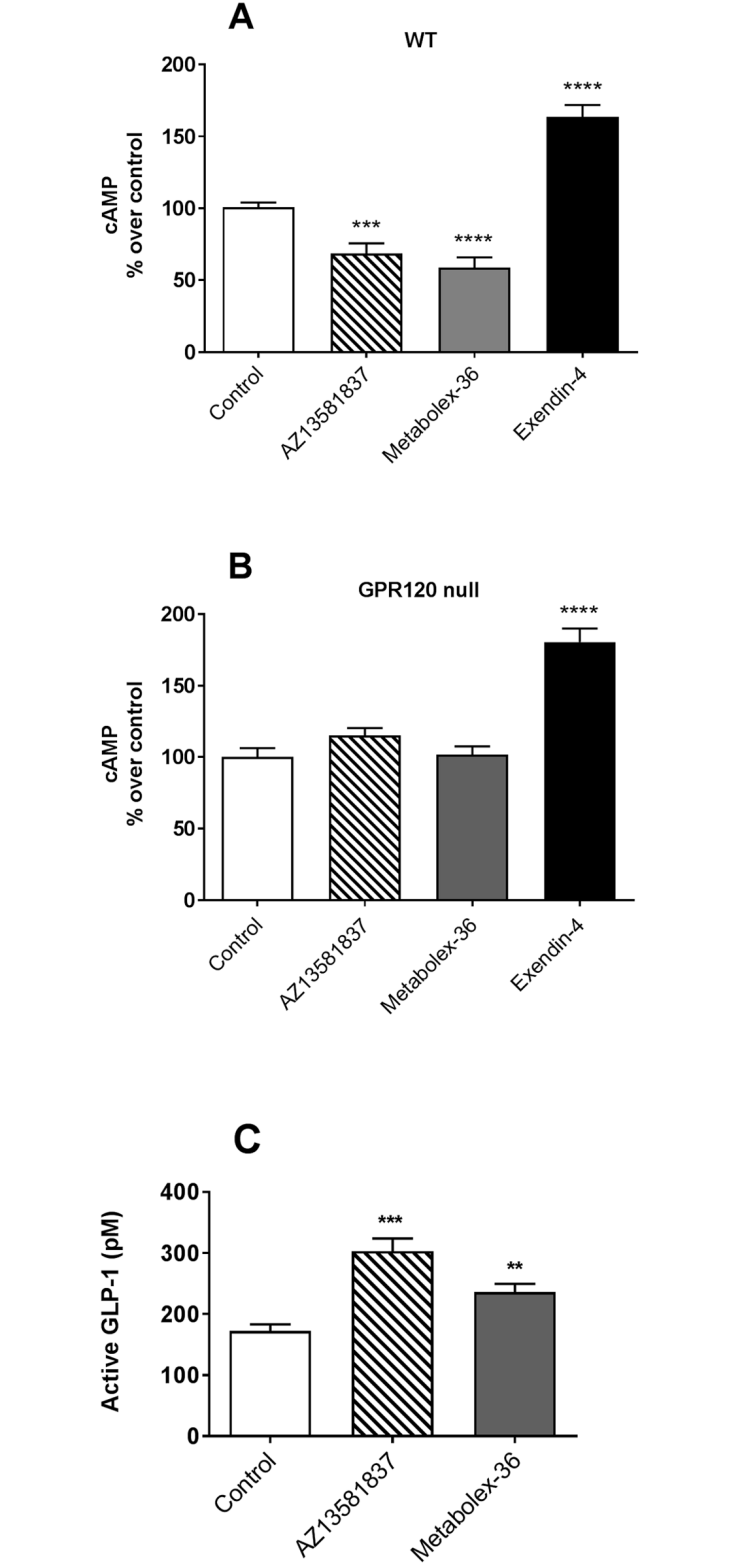
AZ13581837 and Metabolex-36 reduced cAMP production in mouse islets and induced GLP-1 secretion from STC-1 cells. Effect of 10 μM AZ13581837, 10 μM Metabolex-36, 50 nM Exendin-4 or vehicle control on cAMP production in dispersed islets from wild type (**A**) and GP120 *null* mice (**B**). Data represent mean ± SEM from three independent experiments where islet were isolated from two or four mice of each genotype. cAMP was measured in at least triplicates for both wild type and GPR120 *null* islet in each experiment. STC-1 cells were stimulated with Metabolex-36, AZ13581837 or vehicle control (0.1% DMSO) for 2 hours and secreted active GLP-1 was measured by ELISA (**C**). Three independent GLP-1 secretion experiments were run where n = 3 of each control and compound treatment. *p<0.05, **p<0.01, ***p<0.001 and ****p<0.0001 versus vehicle control (two sample, two sided t-test).

To explore GPR120 signalling in a primary cell type we determined the cAMP levels in primary mouse islets. GPR120 is expressed in mouse islets with major expression in δ cells and where activation results in inhibition of somatostatin secretion [26]. To demonstrate specificity, islet cells from both wt and GPR120 *null* mice were examined. Stimulation of dispersed wt mouse islets cell with 10 μM AZ13581837 or Metabolex-36 significantly reduced cAMP production compared to control cells (Fig 3A), whereas no effect was detected in islets from GPR120 *null* mice (Fig 3B). Exendin-4, a GLP-1 receptor agonist which signals through Gα_s_, was used as positive control and it was shown to significantly stimulate cAMP production in islet cells from both wt and GPR120 *null* mice (Fig 3A and 3B).

GPR120 dependent GLP-1 secretion was examined in the enteroendocrine cell line STC-1. Expression of GPR120 in STC-1 cells was verified with gene expression analyses (data not shown). Activation of GPR120 by AZ13581837 or Metabolex-36 significantly increased GLP-1 secretion from STC-1 cells (Fig 3C).

### GPR120 agonists improve oral glucose tolerance in mice

**Fig 4:**
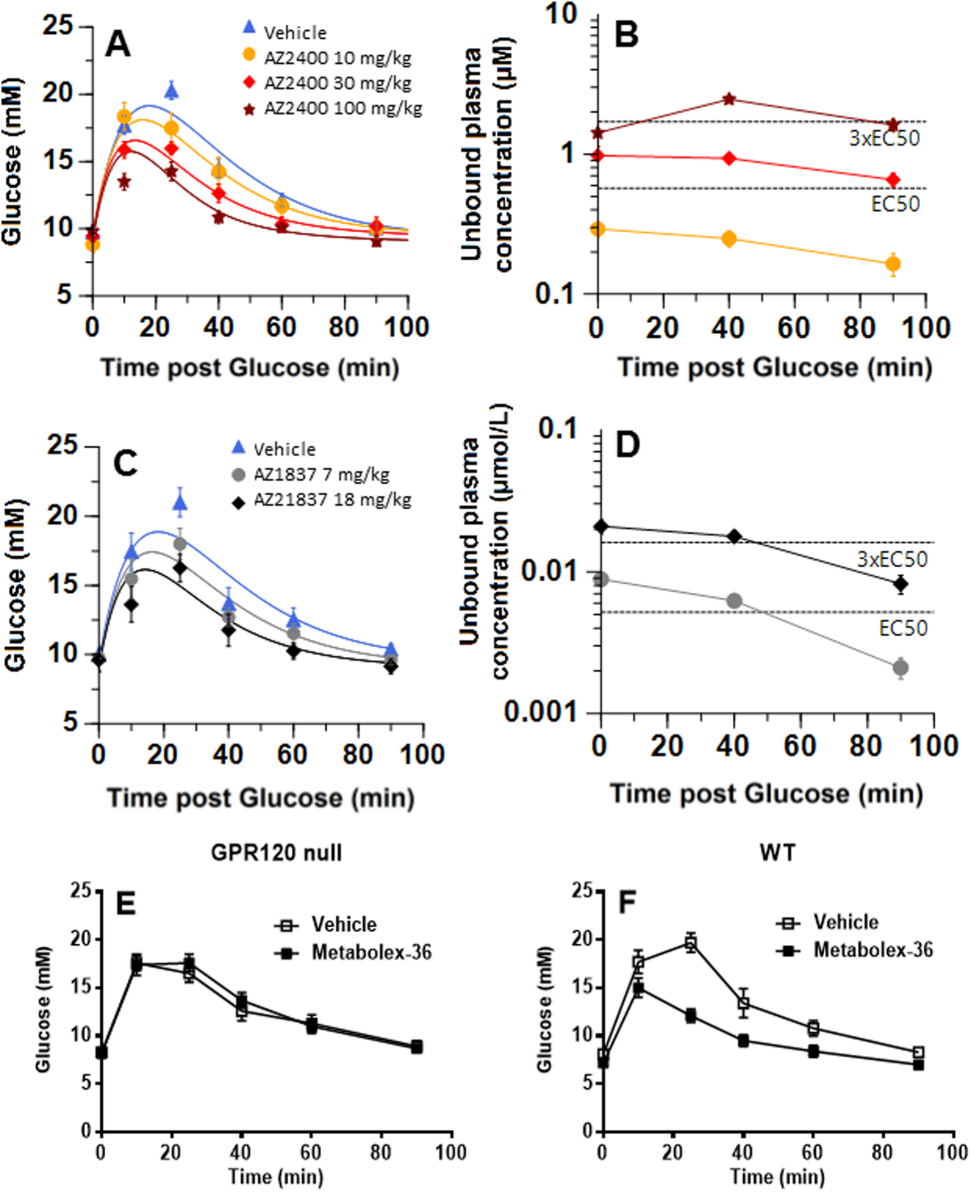
Effect of Metabolex-36 and AZ13581837 on oral glucose tolerance in mice. Effect of Metabolex-36 (**A**) and AZ13581837 (**C**) on glucose response after an oral glucose challenge (2g/kg) in male mice and the corresponding unbound circulating concentrations of Metabolex-36 (**B**) and AZ13581837 (**D**) during the experiment. AZ13581837 and Metabolex-36 were given in different doses as indicated in the figures with n = 10 mice group and compared to vehicle treated mice (n = 12 mice per group). The EC_50_ value for each GPR120 agonist assessed on mouse GPR120 using a DMR assay is indicated in figures. Blood glucose levels following oral glucose administration in GPR120 *null* mice (**E**) and wild type mice (**F**) were determined for vehicle (open squares) and Metabolex-36 (filled squares).

Oral glucose tolerance tests were used to determine the *in vivo* potency of GPR120 agonists and to select doses for *in vivo* studies designed to evaluate the pharmacodynamic effects (S2 Appendix). Administration of Metabolex-36 or AZ13581837 in different doses to lean male mice resulted in a concentration dependent reduction in glucose excursions following oral glucose administration, compared to vehicle treated mice (Fig 4A and 4C). Significantly improved glucose tolerance was observed at 30 mg/kg with Metabolex-36 and at 18 mg/kg for AZ13581837. The calculated unbound effective concentration, Ce_50_ values, were 0.6 μM for Metabolex-36 (Fig 4B), and 0.02 μM for AZ13581837 (Fig 4D), corresponding to exposure levels of 1–3 times *in vitro* EC_50_ (mouse GPR120 DMR assay). The specificity of Metabolex-36 was demonstrated in GPR120 *null* mice, where no effect on oral glucose tolerance of Metabolex-36 could be observed (Fig 4E and 4F).

### GPR120 activation increased the insulin response following intravenous glucose administration in mice

**Fig 5:**
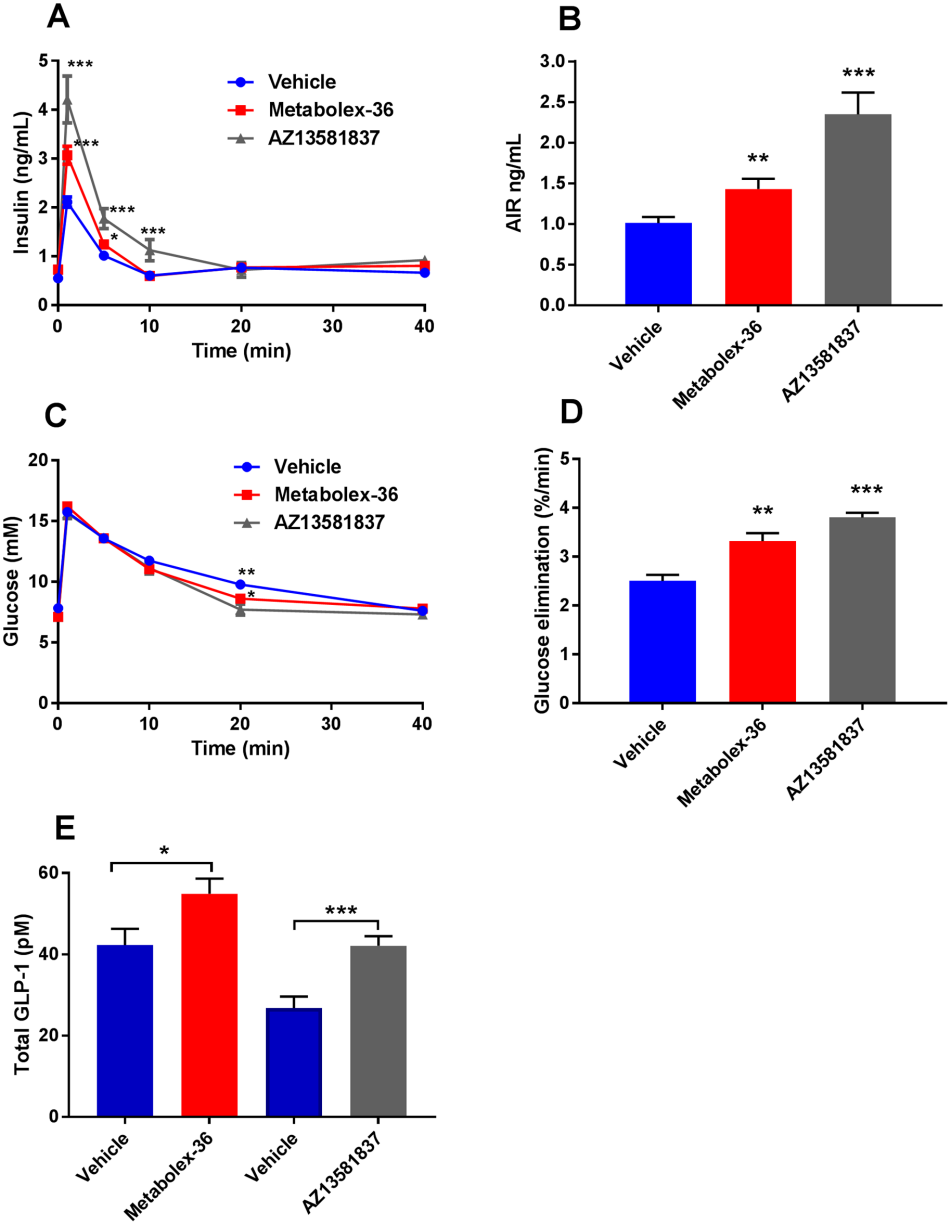
Metabolex-36 and AZ13581837 increased insulin secretion in IVGTT in lean mice. Insulin (**A**) and blood glucose (**C**) levels following an intravenous glucose challenge after oral administration of Metabolex-36 and AZ13581837 in lean female mice and corresponding AIR (**B**) and glucose elimination (**D**). Data represent six (Metabolex-36, n = 33, vehicle n = 34) and two (AZ13581837, n = 14) independent experiments and data are presented as mean ± SEM. Plasma levels of total GLP-1 (**E**) at time point was determined in separate experiments with n = 10 mice per group. ***p<0.001 and **p<0.01versus vehicle control.

Since oral administration of glucose induces endogenous incretin secretion [27], we examined the incretin secretory capacity of GPR120 activation by performing IVGTT in 4-hour fasted female mice. We observed a slight but significant reduction in basal blood glucose with Metabolex-36 (6.9±0.2 mM, p<0.001) and AZ13581837 (7.1±0.3 mM, p<0.001) compared to vehicle treated mice (7.8±0.2 mM). Mice that were pre-administered orally with the GPR120 agonists’ demonstrated significantly elevated insulin levels up to 10 minutes after the iv glucose bolus for AZ13581837, and up to 5 minutes with Metabolex-36 (Fig 5A). Calculation of the acute insulin response, as the mean insulin release during 5 min, showed a significant increase for both agonists compared to the controls, where AZ13581837 produced a more pronounced effect than Metabolex-36 (Fig 5B). The increased insulin secretion resulted in faster elimination of glucose from the blood for both GPR120 agonists with significant difference in plasma glucose 20 min after the glucose load for both compounds (Fig 5C). Glucose elimination was calculated between 1 and 20 min and both GPR120 agonists increased the glucose elimination (Fig 5D).

In separate experiments, total GLP-1 levels were measured 30 or 60 minutes after administration of AZ13581837 or Metabolex-36, respectively. The GLP-1levels were compared to vehicle administered mice, which were slightly different between the two experiments. However, compared to their respective control group, both GPR120 agonists induced a significant increase in total GLP-1 levels, demonstrating increased GLP-1 secretion (Fig 5E).

### GPR120 activation has no effect on insulin secretion in GPR120 *null* mice

**Fig 6:**
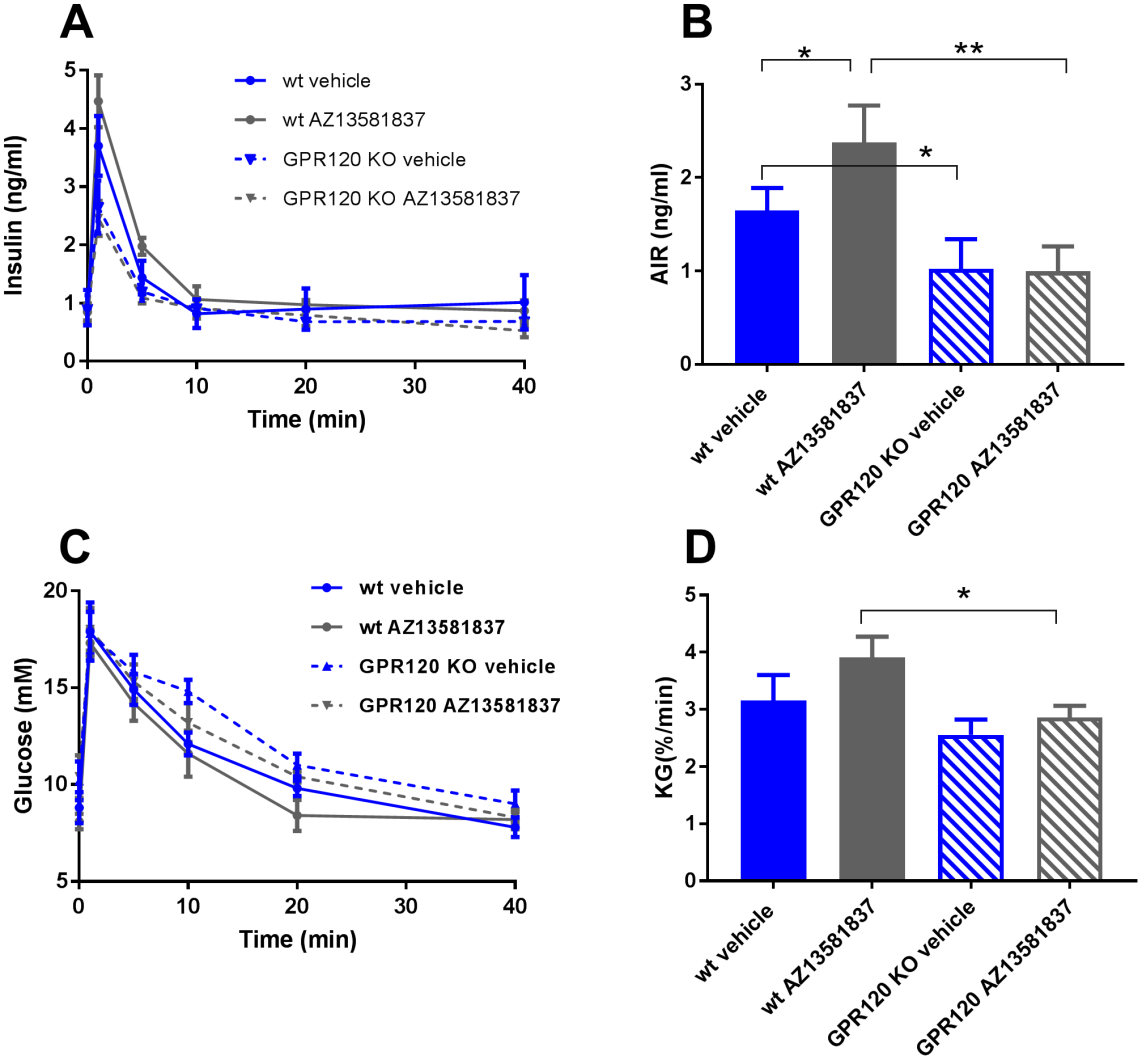
AZ13581837 had no effect on insulin secretion in GPR120 *null* mice. Insulin (**A**) and blood glucose (**C**) levels following an intravenous glucose challenge after administration of AZ13581837 in female, lean wt or GPR120 *null* mice and corresponding AIR (**B**) and glucose elimination (**D**). The results are from one experiment with n = 4 per group. Data are presented as mean ± SEM.**p<0.01, *p<0.05.

We demonstrated that the Metabolex-36 compound had no effect on glucose lowering in male GPR120 *null* mice following oral glucose administration (Fig 4E). To evaluate the specificity of our GPR120 agonist, AZ13581837, and demonstrate effects on insulin secretion, IVGTT was performed in female wild type (wt) and GPR120 *null* mice. Similar to previous experiments, AZ13581837 was administered 30 min prior to the intravenous glucose bolus. We found that the compound significantly increased the acute insulin secretion in the wt mice compared to the vehicle group (p<0.05) while there was no effect observed in GPR120 *null* mice compared to vehicle treated control mice of respective genotype (Fig 6A and 6B). The acute insulin response in the GPR120 *null* mice was reduced *per se* compared to wt mice treated with vehicle (p<0.05) or wt mice treated with AZ13581837 (p<0.01, Fig 6B). The reduced insulin response was reflected as impaired glucose elimination in the GPR120 *null* mice and no effect of AZ13581837 (Fig 6D). The effect of glucose elimination by AZ13581837 in wt mice was increased compared to compound treated GPR120 *null* mice (p<0.05, Fig 6D).

### The glucose lowering effect of GPR120 agonists *in vivo* is dependent on GLP-1

**Fig 7:**
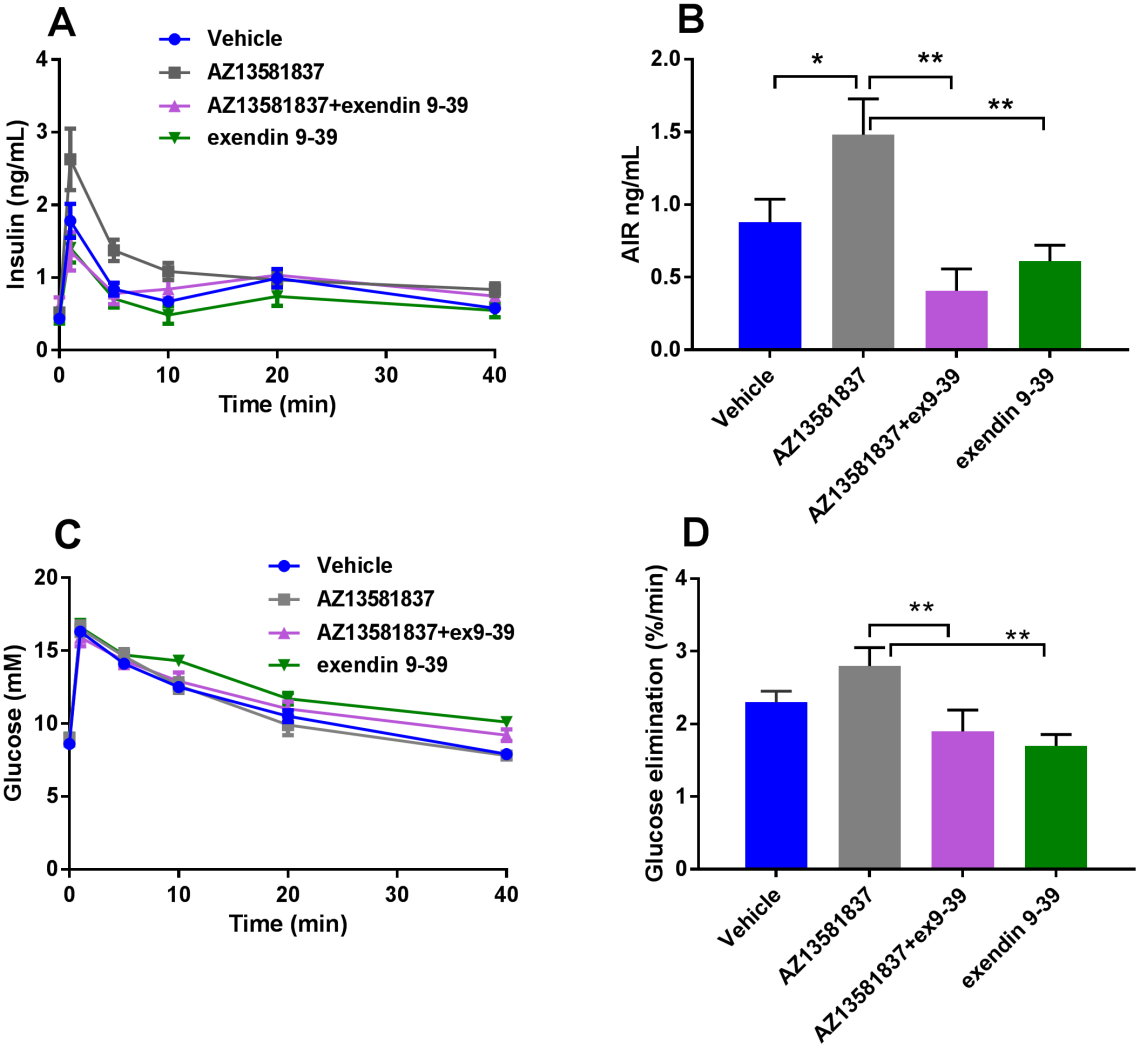
Exendin 9–39 blocked the AZ13581837 induced potentiation of insulin secretion in lean mice. Insulin levels following intravenous glucose challenge (**A**) and corresponding blood glucose (**C**), after administration of AZ13581837, exendin 9–39 or a co-administration of both, with corresponding calculations of AIR (**B**) and glucose elimination (**D**). The IVGTT data are from two independent experiments with n = 10 mice per group. Data are presented as mean ± SEM.***p<0.001 and *p<0.05 versus vehicle control.

To demonstrate the importance of GLP-1 for the acute effects on insulin secretion induced by GPR120 agonism, we pretreated mice with the GLP-1 receptor antagonist, exendin 9–39. Exendin 9–39 was given after dosing with the GPR120 agonist but 15 min before the iv glucose bolus. As in earlier experiments, AZ13581837 produced a significant increase in insulin secretion (Fig 7A) which was completely abolished after co-administration with exendin 9–39 (Fig 7A). The acute insulin response was similar to vehicle mice when AZ13581837 was give together with exendin 9–39 (Fig 7B). Exendin 9–39 alone had no significant effect on insulin secretion, although there was a trend towards lower response compared to the vehicle group with both exendin 9–39 groups (Fig 7B). As expected, glucose elimination was similar to vehicle treated mice when exendin 9–39 was given alone or together with AZ13581837 and significantly reduced compared to AZ 13581837 alone (Fig 7C and 7D).

**Fig 8:**
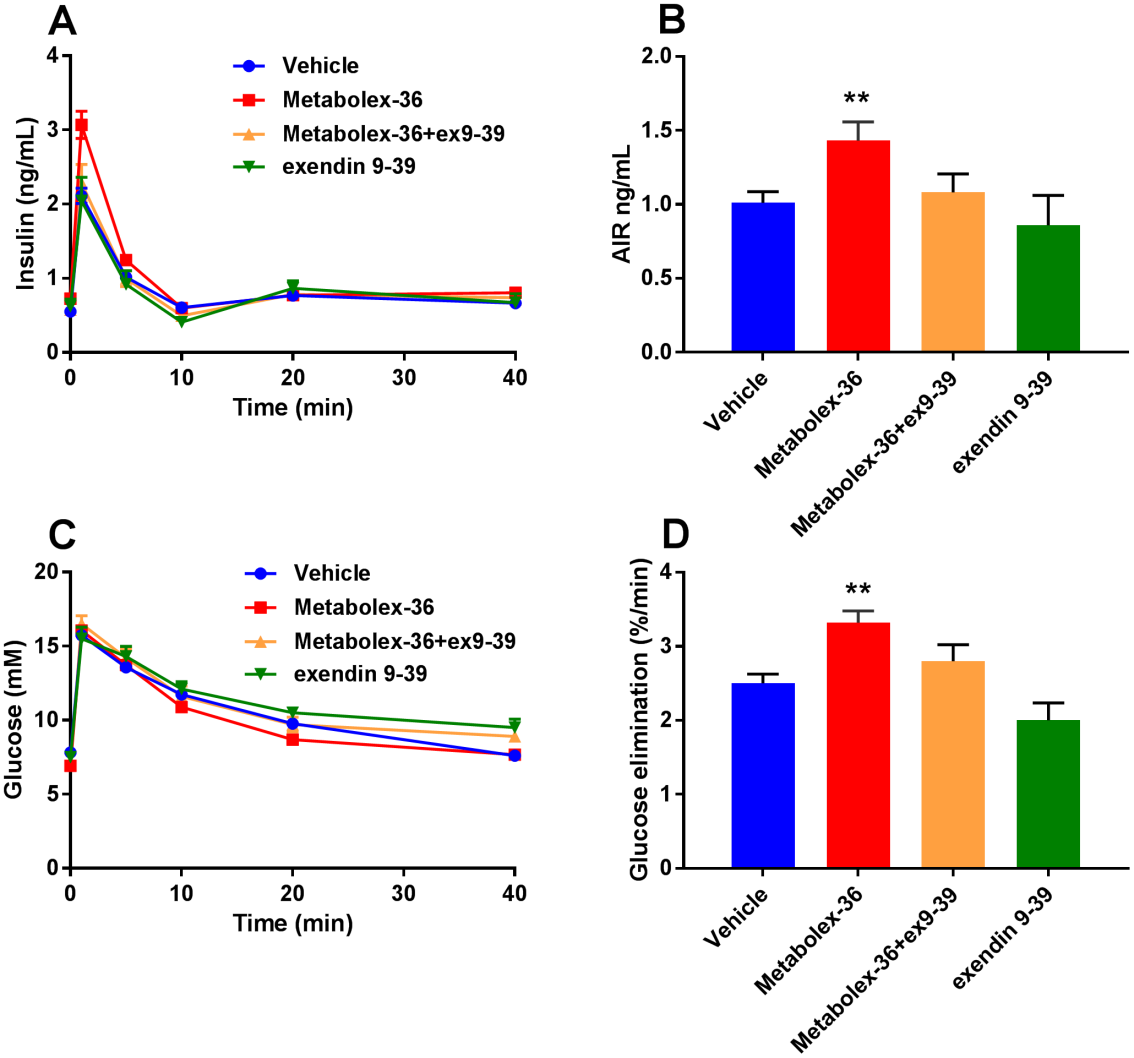
Exendin 9–39 blocked the Metabolex-36 induced potentiation of insulin secretion in lean mice. Insulin levels following intravenous glucose challenge (**A**) and corresponding blood glucose (**C**), after administration of Metabolex-36, exendin 9–39 or a co-administration of both, with corresponding calculations of AIR (**B**) and glucose elimination (**D**). The IVGTT data are from two independent experiments with 6–7 mice per group. Data are presented as mean ± SEM.**p<0.01 and *p<0.05 versus vehicle control.

Similar results were obtained with Metabolex-36, where the increased insulin response as well as the glucose lowering effect were abolished when administered in combination with exendin 9–39 (Fig 8A and 8C). Similarly, the acute insulin response and the glucose elimination were reduced to vehicle levels by exendin 9–39 (Fig 8B and 8D). Also in this experiment, exendin 9–39 had no significant effect on insulin secretion by itself, although again a trend towards lower levels of insulin and reduced glucose elimination were observed (Fig 8B and 8D).

## Discussion

Glucose homeostasis is regulated by multiple signals including the key hormones insulin and glucagon secreted from the pancreatic islets and GLP-1 from the gut enteroendocrine L-cells. GPR120, which is expressed in both islet and in enteroendocrine cells, is activated by medium- and long-chain fatty acids (LCFAs) and has been extensively investigated as a drug target for treatment of obesity and type 2 diabetes due to its many effects on metabolism including increasing secretion of GLP-1 [28, 29]. However, the exploration of this receptor as a therapeutic target has been limited by the lack of potent and selective agonist ligands without activity on GPR40 and only recently selective agonists have started to emerge [30–34]. We have developed a novel GPR120 specific agonist, AZ13581837, and studied its pharmacology and role in glucose metabolism in comparison to a previously published GPR120 agonist, Metabolex-36 [19].

Activation of GPR120 by these two selective agonists induced calcium mobilization and β-arrestin recruitment in GPR120 overexpressing cells as previously observed in other studies with LCFAs [4, 35], non-selective [16, 36–38] and selective small molecules [34]. *In vitro* comparison with the GPR120 selective small molecule cpdA (EC_50_ 0.35 μM in β-arrestin and 24 nM in calcium mobilization) [34], AZ13581837 was similar being more potent on β-arrestin but less potent in calcium mobilization, while Metbaolex-36 was much less potent in both assays (Table 1). Besides the commonly described Gα_q_ and β-arrestin mediated pathways, we found that GPR120 also signals through Gα_s_, increasing cAMP in CHO cells overexpressing the human GPR120 receptor. In CHO cells overexpressing the mouse GPR120 receptor we were however unable to detect any AZ13581837 or Metabolex-36 dependent activation or inhibition of cAMP production (data not shown). A previous study in pancreatic BRIN-BD11 beta cells suggested that activation of GPR120 stimulated cAMP production [39] and recently GPR120 was suggested to be involved in inhibition of GLP-2 production in L cells via the Gα_s_ pathway [40]. However, both studies used dual GPR120 and GPR40 agonists and since activation of GPR40 has been reported to induce Gα_s_ signaling and cAMP production [41] it is difficult to draw conclusions. We did not detect any Gα_i_ coupling to GPR120 in overexpressing CHO cells, which is in contrast to a previous study [42], demonstrating inhibition of ghrelin secretion in gastric ghrelin expressing cells after activation of GPR120 through the Gα_i_-pathway. However, we could demonstrate that activation of GPR120 in mouse islets resulted in inhibited production of cAMP, illustrating the importance of the cellular context when examining signaling pathways and physiological importance. Thus to our knowledge, our data are the first to demonstrate effects on cAMP production with selective GPR120 agonists. The mechanisms and importance of biased signaling of GPR120 has started to be explored [43, 44]. Different signaling pathways in different cell types were demonstrated with GW9508 and TUG-891 that recruited β-arrestin-2 in Caco-2 cells but not in STC-1 cells [43]. However, to fully elucidate the biological function of GPR120, the generation of selective biased agonists and assessment of signaling in different cell types are critical [45].

The role of GPR120 for stimulation of GLP-1 secretion has been well established *in vitro* [4] and was confirmed in this study in STC-1 cells, while the contribution of GPR120 activation *in vivo* for GLP-1 secretion is not completely elucidated. Species selectivity is a known issue for the GPR120 agonists [46] and it is only recently that compounds suitable for rodent models have been described [30–33]. AZ13581837 and Metabolex-36 were well suited for acute *in vivo* studies in mice. Both have similar activities on mouse and human GPR120, and no activity on mouse or human GPR40, although with insufficient pharmacokinetic properties (e.g. short half-life) to be used for chronic treatment.

Sensing of fat via the fatty acid receptors in the gut has been extensively investigated *in vivo* [47] and GPR120 has been suggested as the main pathway for the lipid-induced GLP-1 secretion [4]. However, a recent study presented that ALA had no significant effect on active GLP-1 levels [17]. In the present study, we demonstrate that oral administration of GPR120 specific agonists caused a significant increase in total GLP-1 levels in lean mice. Total GLP-1 levels represent a better measure of the secretion rate, since GLP-1 is rapidly degraded in the circulation. However, another recent study suggested that GPR40 is the main pathway for GLP-1 secretion from the gut since corn oil was unable to induce GLP-1 secretion in GPR40 *null* mice while the effect was maintained in GPR120 *null* mice [48], proposing that GPR120 only plays a minor role in GLP-1 release. Taken together, species differences and various types of fatty acids may play different roles in the incretin release, and it is only when using selective and specific agonists that the mechanism of a GPR120 can be fully explored.

In our study we used selective GPR120 agonists and subjected the mice to intravenous glucose challenge to avoid interference of incretin effects from administration of glucose in the gut. The GPR120 agonists significantly reduced basal blood glucose and this correlated with elevated GLP-1 levels. After the glucose bolus, insulin secretion was increased and this correlated to faster elimination of glucose. Exendin 9–39 is a potent and specific GLP-1 receptor antagonist, which is frequently used to demonstrate effects induced by endogenously produced GLP-1 [49–51]. Both in humans and in rodents, exendin 9–39 efficiently blocks the effect of GLP-1 on insulin secretion and it has even been shown to induce hyperglycemia despite a compensatory increase in endogenous GLP-1 levels [49, 50]. These data support that exendin 9–39 is a good tool to explore GLP-1 mediated effects *in vivo*. When we pre-treated mice with exendin 9–39, 15 minutes prior to the glucose load but after administration of the GPR120 agonists, the GPR120 mediated increase in glucose-stimulated insulin secretion was completely abolished and both insulin and glucose levels were similar to vehicle treated mice. This demonstrates that GLP-1 secretion, which is increased following GPR120 activation, contribute significantly to the glucose lowering effect, although it cannot be excluded that other mechanisms may also be involved. For example, GPR120 is also expressed in islets and activation may indirectly affect insulin secretion. In a previous study in mice, fatty acids or GW-9508 were administered together with glucose and the observed elevation of insulin section was suggested to be mediated via GPR120 on beta cells [39]. Again, it cannot be ruled out that the observed effects at least partially occurred through activation of GPR40. Fatty acids potentiate glucose-stimulated insulin secretion mainly via activation of GPR40, which is abundantly expressed in beta cells, whereas GPR120 is preferentially expressed in delta cells in rodent islets [26]. Metabolex-36 has been shown to reduce somatostatin secretion *in vitro* from mouse islets [26] and since somatostatin is a potent inhibitor of insulin secretion, this is another possible mechanism that may contribute to increasing insulin release following GPR120 activation. The observed reduction in somatostatin secretion will likely occur through Gα_i_ coupling since preincubation of mouse islets with PTX significantly attenuated the inhibitory effects of Metabolex-36 on glucose-induced somatostatin secretion [26]. This is supported by our finding that the GPR120 activation resulted in inhibition of cAMP in mouse islets, which is expected to occur through Gα_i_. A recent study highlights the complexity and suggest that the islet may play an even more important role, where the islet produced GLP-1 was found to be a significant player in the glucose homeostasis in mice [51].

## Conclusions

We have developed a selective and specific GPR120 agonist suitable for acute *in vivo* studies in mice and demonstrated that one important mechanism behind the acute glucose lowering effect of specific GPR120 activation is dependent on GLP-1. We also show that GPR120 signals though different G-proteins in different cell types and we demonstrate that in primary mouse islet cells, GPR120 reduce cAMP levels, suggesting involvement of Gαi signalling.

## Supporting information

S1 Appendix Synthesis of 2-[3-ethynyl-5-(3-pyridyloxy)phenyl]-3H-1,2-benzothiazole 1,1-dioxide (AZ13581837). (DOCX)

S2 Appendix *In vitro* and *in vivo* relationship to build dose predictions. (DOCX)
