# Supplementary material for: The acute glucose lowering effect of specific GPR120 activation in mice is mainly driven by glucagon-like peptide 1
Source: PLoS One. 2017 Dec 5;12(12):e0189060. doi: 10.1371/journal.pone.0189060 (PMC5716539; doi:10.1371/journal.pone.0189060)
Supplement: S1 Appendix — (DOCX) [file pone.0189060.s001.docx]

**S1 Appendix**

**Synthesis of 2-[3-ethynyl-5-(3-pyridyloxy)phenyl]-3H-1,2-benzothiazole 1,1-dioxide (AZ13581837)**.

1,3-dibromo-5-fluorobenzene (3.44 g, 13.6 mmol), pyridin-3-ol (1.42 g, 14.9 mmol) and potassium carbonate (4.68 g, 33.9 mmol) were suspended in dimethylformamide (DMF) (40 ml) and sealed into a microwave tube. The reaction was heated to 140°C for 4 hours in the microwave reactor and cooled to room temperature. The reaction mixture was diluted with EtOAc (75 ml), washed with water (75 ml), and saturated brine (75 ml). The organic layer was dried (MgSO_4_), filtered, evaporated and purified by flash silica chromatography, elution gradient (10 to 60% EtOAc in isohexane) to afford 3-(3,5-dibromophenoxy)pyridine (1.84 g, 41%) as a colourless oil. ^1^H NMR (400 MHz, CDCl_3_, 30°C) 7.09 (2H, d), 7.3 - 7.38 (2H, m), 7.44 (1H, t), 8.41 - 8.44 (1H, m), 8.45 - 8.5 (1H, m); m/z MH^+^ = 328, 330, 332.

Palladium(II) acetate (0.062 g, 0.28 mmol) was added to 3-(3,5-dibromophenoxy)pyridine (1.83 g, 5.56 mmol), benzophenone imine (0.933 ml, 5.56 mmol), cesium carbonate (2.18 g, 6.68 mmol) and rac-2,2'-bis(diphenylphosphino)-1,1'-binaphthyl (0.173 g, 0.28 mmol) in degassed THF (20 ml) under nitrogen. The resulting mixture was stirred at 100°C in the microwave for 6 hours, cooled to room temperature, diluted with diethyl ether (30 ml) and filtered. The filtrate was evaporated and purified by flash silica chromatography, elution gradient (0 to 20% EtOAc in heptanes), to afford 3-bromo-N-(diphenylmethylene)-5-(pyridin-3-yloxy)aniline (1.64 g, 69%) as a yellow gum. ^1^H NMR (400 MHz, CDCl_3_, 30°C) 6.17 (1H, t), 6.78 (2H, dt), 6.94 - 7.03 (1H, m), 7.09 (2H, dd), 7.14 - 7.21 (1H, m), 7.29 - 7.43 (5H, m), 7.49 (1H, dd), 7.66 - 7.77 (2H, m), 8.21 (1H, d), 8.34 (1H, dd); m/z MH^+^ = 429, 431.

2 M aqueous HCl (5.73 ml, 11.5 mmol) was added to 3-bromo-N-(diphenylmethylene)-5-(pyridin-3-yloxy)aniline (1.64 g, 3.82 mmol) in tetrahydrofuran (THF) (40 ml) at 20°C. The resulting mixture was stirred at 20°C for 16 hours, quenched with 2N Na_2_CO_3_ (50 ml), and extracted with dichloromethane (DCM) (2 x 75 ml). The organic layers were combined, evaporated and purified by flash silica chromatography, elution gradient (20 to 80% EtOAc in isohexane), to afford 3-bromo-5-(pyridin-3-yloxy)aniline (0.76 g, 75%) as a colourless oil. ^1^H NMR (400 MHz, CDCl_3_, 30°C) 3.83 (2H, s), 6.23 (1H, t), 6.49 - 6.53 (1H, m), 6.59 - 6.62 (1H, m), 7.27 - 7.35 (2H, m), 8.37 - 8.43 (2H, m); m/z MH^+^ = 265, 267.

Methyl 2-(chlorosulfonyl)benzoate (0.896 g, 3.44 mmol) was added in one portion to 3-bromo-5-(pyridin-3-yloxy)aniline (0.759 g, 2.86 mmol) in pyridine (15 ml) under nitrogen. The resulting solution was stirred at room temperature for 2 hours, evaporated, diluted with water (50 ml) and saturated NH_4_Cl (50 ml), then extracted with EtOAc (2 x 100 ml). The combined organics were washed with 1 M citric acid (50 ml) and saturated brine (50 ml). The organic layer was dried (MgSO_4_), filtered, evaporated and purified by flash silica chromatography, elution gradient (10 to 60% EtOAc in heptanes), to afford methyl 2-(N-(3-bromo-5-(pyridin-3-yloxy)phenyl)sulfamoyl)benzoate (0.96 g, 73%) as a white solid. ^1^H NMR (400 MHz, CDCl_3_, 30°C) 4.03 (3H, s), 6.87 (2H, dt), 7.09 (1H, t), 7.21 - 7.26 (1H, m), 7.27 - 7.32 (1H, m), 7.62 (2H, dtd), 7.88 (2H, ddd), 8.18 (1H, s), 8.29 (1H, d), 8.42 (1H, dd); m/z MH^+^ = 463,465.

Lithium aluminum hydride (2M in THF) (1.04 ml, 2.07 mmol) was added dropwise to methyl 2-(N-(3-bromo-5-(pyridin-3-yloxy)phenyl)sulfamoyl)benzoate (0.96 g, 2.07 mmol) in THF (30 mL) at 0°C under nitrogen. The resulting solution was stirred at 0°C for 2 hours. The reaction mixture was quenched with 2 M NaOH (0.1 ml) and water (0.3 ml), filtered to remove aluminum salts and evaporated. Ethyl acetate (100 ml) was added and washed with water (50 ml) and brine (50 ml), dried (MgSO_4_), filtered and evaporated to afford a colourless gum. The residue from the filtration was stirred in EtOAc (100 ml) and a solution of Rochelle's salt (100 ml) and the ethyl acetate layer separated, dried (MgSO_4_), filtered and evaporated to afford more product which was combined to afford N-(3-bromo-5-(pyridin-3-yloxy)phenyl)-2-(hydroxymethyl)benzenesulfonamide (0.84 g, 93%) as a colorless gum. ^1^H NMR (400 MHz, CDCl_3_, 30°C) 5.10 (2H, s), 6.73 (1H, t), 6.84 (1H, t), 7.00 (1H, t), 7.21 (1H, ddd), 7.26 - 7.31 (1H, m), 7.42 (2H, dd), 7.54 (1H, td), 7.74 - 7.84 (1H, m), 8.24 (1H, d), 8.39 (1H, dd); m/z MH^+^ = 435,437.

Diisopropylazodicarboxylate (0.417 ml, 2.12 mmol) was added to N-(3-bromo-5-(pyridin-3-yloxy)phenyl)-2-(hydroxymethyl)benzenesulfonamide (0.838 g, 1.93 mmol) and triphenylphosphine (0.555 g, 2.12 mmol) in THF (25 ml) at 0°C under nitrogen. The resulting mixture was allowed to warm to room temperature and stirred for 3 days. The reaction mixture was evaporated to dryness and redissolved in EtOAc (75 ml), washed with water (50 ml) and saturated brine (50 ml). The organic layer was dried (MgSO_4_), filtered, evaporated and purified by flash silica chromatography, elution gradient (10 to 40% EtOAc in heptane followed by 1-4% MeOH in DCM), to afford 2-[3-bromo-5-(3-pyridyloxy)phenyl]-3H-1,2-benzothiazole 1,1-dioxide (0.53 g, 66%) as a white solid. ^1^H NMR (400 MHz, CDCl_3_, 30 °C) 4.84 (2H, s), 6.88 - 6.94 (1H, m), 7.15 (1H, t), 7.3 - 7.42 (3H, m), 7.50 (1H, d), 7.62 (1H, t), 7.70 (1H, td), 7.88 (1H, d), 8.41 - 8.5 (2H, m); m/z MH^+^ = 417,419.

2-[3-Bromo-5-(3-pyridyloxy)phenyl]-3H-1,2-benzothiazole 1,1-dioxide (0.15 g, 0.36 mmol) was added to dichlorobis(triphenylphosphine)palladium(II) (0.050 g, 0.07 mmol) and copper(I) iodide (0.021 g, 0.11 mmol) and the reaction vessel was evacuated and purged with nitrogen. THF (3 ml) and ethynyltrimethylsilane (0.071 g, 0.72 mmol) were added under nitrogen. The resulting mixture was stirred and then triethylamine (0.100 ml, 0.72 mmol) was added and the resulting mixture was stirred at room temperature for 3 hours, evaporated to dryness, redissolved in EtOAc (100 ml), and washed with water (100 ml), saturated brine (100 ml) and filtered through celite^®^. The celite^®^ was washed with DCM/MeOH and the organic layers combined, dried (MgSO4), filtered, evaporated and purified by flash silica chromatography, elution gradient (10 to 50% EtOAc in isohexane), to afford 2-[3-(1,1-dioxo-3H-1,2-benzothiazol-2-yl)-5-(3-pyridyloxy)phenyl]ethynyl-trimethyl-silane (0.044 g, 28%) as a yellow gum. ^1^H NMR (400 MHz, CDCl_3_, 30 °C) 0.24 (9H, s), 4.85 (2H, s), 6.8 - 6.87 (1H, m), 7.21 (1H, t), 7.27 - 7.41 (3H, m), 7.50 (1H, d), 7.61 (1H, t), 7.69 (1H, t), 7.87 (1H, d), 8.4 - 8.44 (1H, m), 8.44 - 8.48 (1H, m); m/z MH^+^ = 435.

Sodium hydroxide (2 M in water) (0.127 ml, 0.25 mmol) was added to 2-[3-(1,1-dioxo-3H-1,2-benzothiazol-2-yl)-5-(3-pyridyloxy)phenyl]ethynyl-trimethyl-silane (0.044 g, 0.10 mmol) in methanol (2 ml). The resulting solution was stirred at room temperature for 30 min, evaporated then diluted with EtOAc (100 ml), washed with water (50 ml), and saturated brine (50 ml). The organic layer was dried (MgSO_4_), filtered, evaporated and purified by flash silica chromatography, elution gradient (20 to 80% EtOAc in isohexane), to afford the title compound 2-[3-ethynyl-5-(3-pyridyloxy)phenyl]-3H-1,2-benzothiazole 1,1-dioxide (AZ13581837) (0.030 g, 82%) as a white solid. ^1^H NMR (400 MHz, CDCl_3_, 30 °C) 3.12 (1H, s), 4.85 (2H, s), 6.87 (1H, dd), 7.23 (1H, t), 7.28 - 7.39 (3H, m), 7.50 (1H, d), 7.61 (1H, t), 7.70 (1H, td), 7.88 (1H, d), 8.43 (1H, dd), 8.46 (1H, d); ^13^C NMR (101 MHz, CDCl_3_) 49.4, 78.8, 82.3, 109.9, 117.2, 117.2, 121.7, 124.3, 124.5, 125.0, 126.1, 129.7, 131.5, 133.4, 134.9, 139.0, 142.0, 145.3, 152.8, 157.5; m/z MH^+^ = 363; HRMS (ES^+^) for C_20_H_15_N_2_O_3_S (MH^+^): calculated, 363.07979; found, 363.07983.
