# Supplementary material for: The acute glucose lowering effect of specific GPR120 activation in mice is mainly driven by glucagon-like peptide 1
Source: PLoS One. 2017 Dec 5;12(12):e0189060. doi: 10.1371/journal.pone.0189060 (PMC5716539; doi:10.1371/journal.pone.0189060)
Supplement: S2 Appendix — (DOCX) [file pone.0189060.s002.docx]

**S2 appendix**

**In vitro and in vivo relationship to build dose predictions**

To build a relationship between *in vitro* and *in vivo* effects of GPR120 agonists a single point OGTT test was performed in lean male mice. An oral glucose load (2 g/kg) was administered 30 or 60 min after dosing with GPR120 agonists and blood glucose was measured 15 min after the glucose load. One group of animals were administered only water to establish a baseline glucose level. An E_max_ model (equation 1), where IC_50_ denotes half maximum effect, I_max_ denotes maximum effect, n denotes the slope factor and C denotes the concentration, was fitted to the data from multiple compounds taking into consideration the *in vitro* potency (EC_50_) from the DMR assay and protein binding (f_u_). The baseline glucose level was set as the maximum inhibition achievable.

$E=\frac{I_{max}\times{{(C\times f_{u}}/{{EC}_{50})}}^{n}}{{{IC}_{50}}^{n}+{{(C\times f_{u}}/{{EC}_{50})}}^{n}}$ (1)

The model was used to support the prediction of relevant doses of other GPR120 agonists for more detailed *in vivo* studies and suggested a target level of approximately 10 times unbound exposure over in vitro EC_50_ to reach robust effects *in vivo*. The single point OGTT model suggests higher exposure levels for *in vivo* effects than later shown in full time course OGTT, likely due to a less rich data set.

**S2** Fig 1 Single point OGTT glucose levels versus unbound concentration corrected for *in vitro* potency. Several compounds in the series were used to establish a relationship. Dots represent experimental results; the thick line represents model average and the thin lines shows 95% confidence interval of model.
